# Mozart’s or Ambient Music do not Affect Autoalgometric Pain Threshold

**Published:** 2020-05-31

**Authors:** N Vecchione, L Lorusso, A Viggiano

**Affiliations:** Dept. Medicine, Surgery and Dentistry “Scuola Medica Salernitana”, University of Salerno, Italy

**Keywords:** music, pain threshold, autoalgometry

## Abstract

Nowadays, researchers and clinicians are increasingly interested in alternative non-pharmacological treatments, and music therapy seems to have additional and powerful effects on different pathologies and pain. However, since pain is a subjective perception, it is difficult to evaluate if and which effect music has on it.

In this study, a new device and method have been introduced to objectively estimate pain threshold and its changes related to external stimuli. The above-mentioned device, called autoalgometer, allows to evaluate pain threshold changes while listening to music or other sounds.

In this experiment, the pain threshold was evaluated in twenty-seven volunteers after listening to one out of three different soundtracks: white noise, Mozart’s sonata K448 or Brian Eno’s ambient music.

Compared to staying in silence, listening to the recordings had no significant effect on pain threshold, and the results did not show any significant difference between the experimental groups.

Probably, the positive effect of music described in other studies can be ascribed to a psychological effect, meaning that music can improve subjective mood and, thus, modify pain perception.

## I. INTRODUCTION

Pain transmission from primary afferent neurons is modulated by many molecular mechanisms including serotonin [1], opioid system, endocannabinoid system [2], GABA [3], and reactive oxygen species [4,5]. These molecular pathways are targets of several present drugs and for the research on future possible pharmacological treatments of pain.

However, in recent years, the interest in alternative non-pharmacological treatments is growing, and music therapy, in particular, is reporting many interesting clinical results. Several studies, for example, report that the “Mozart effect” is able to decrease both interictal EEG discharges [6–8] and recurrence of clinical seizures [9–14]. Recently, a randomized, open label study confirmed that music therapy may be an additional and effective treatment for patients with refractory epileptic seizures in childhood, and also that a set of different Mozart’s compositions can improve children’s behaviour, including less irritability, self/hetero aggression, tearfulness, mood instability and a better sleep quality [15].

Music seems also useful during blood sampling in premature infants, as demonstrated by significant amelioration of infants’ facial expression of pain after the procedure and by heart rate change during needle extraction; this effect has been achieved through a combination of real womb sounds and sounds heard by the foetus as his/her mother sings, recorded from the uterus of a pregnant woman [16].

Another study reported that music can improve nausea and pain in patients undergoing chemotherapy followed by autologous stem cells transplantation; the patients who received music therapy in this study used significantly less sedative drug (morphine) compared to the no-music therapy patients [17].

Music effect has been investigated during endoscopy and colonoscopy: the aim was to add music therapy to sedation; it has been reported a decrease in anxiety and Propofol consumption and an increased satisfaction in patients who had listened to their favourite music during the procedure [18].

Despite there are studies on pain, it is not easy to evaluate the music effect on pain, because pain is a subjective experience in nature and, thus, complex to quantify. Indeed, the above cited studies have subjective methods to quantify pain intensity, such as using questionnaires, visual analogue scales or numerical rating scales.

Our research group has introduced a new device (autoalgometer) and method (autoalgometry) to objectively estimate pain threshold and its changes associated with external stimuli [19]. This method has been proven to reveal changes in pain threshold due to hypertension [19, 20] or auricular acupressure [21].

Thus, the autolgometer allows to evaluate pain threshold changes while listening to music or other types of sound.

We conducted this research in order to evaluate if and how listening to music can modify pain threshold.

## II. MATERIALS AND METHODS

Twenty-seven healthy volunteers participated to the study; they were 13 women and 14 men of age comprised between 19–31y. Among the participants three were left-handed. All procedures conformed to the directives of the Declaration of Helsinki and an informed consent was obtained by each participant.

Each participant was asked to participate to three experimental sessions spaced at least seven days from each other. In each experimental session, the volunteer listened to one of the following three soundtracks: 1) white noise, 2) Mozart’s sonata K448, 3) Brian Eno’s ambient music (“Music for Airport”). Each soundtrack lasted 4 min. Thus, there were 6 possible sequences of the soundtracks over the three sessions; each participant was pseudo-randomly assigned to one of these sequences, in order to obtain an equal number of participant for each sequence. This design was used to avoid any effect of the sequence on the subsequent analysis of the data.

Since no ideal music characteristics for the management of pain have been yet identified [22], the soundtracks were chosen for the following reasons: Mozart’s sonata K448 was chosen because it is a typical example of music with well defined rhythm and melody and, in previous experiments, our group verified that it had a significant effect for seizure amelioration [14–15]; ambient music was chosen because it completely lacks any rhythm and melody; white noise was chosen as a control acoustic stimulus lacking any musical character.

Three autoalgometer evaluations were taken on each experimental session: 1) at the beginning of the experimental session (basal value); 2) after listening for 4 min to a soundtrack or after staying in silence for 4 min (the condition was randomly chosen for each participant); 3) after staying in silence for 4 min (of after listening for 4 min to a soundtrack if a silence condition was chosen at point 2). Listening to soundtrack or silence continued also during the autoalgometric test (which lasted roughly 5 min).

The autoalgometer evaluation was conducted as previously described [23]; briefly, it consisted of a device with a rounded needle (1 mm in diameter) on which the volunteer had to press his fingers, both the finger tips and backs (the test was repeated on a total of 8 points). For each test, he had to slowly increase the pressure until he felt a minimum or a maximum pain sensation; thus the test was first repeated on the 8 test points to evaluate the minimal pain threshold and then it was repeated on the same 8 test points to evaluate the maximal pain threshold. Because the test speed can affect the measure, pain threshold values were retained for subsequent analyses only when obtained with a mean rate of strength increase lower than 0.5 kg/s [23].

To evaluate the effect of music or silence on pain threshold, the values for the minimal and maximal pain thresholds were expressed as percentage of the basal values taken in the same experimental session.

Statistical evaluation of data was done by the analysis of variance (ANOVA).

## III. RESULTS

**Fig. 1:**
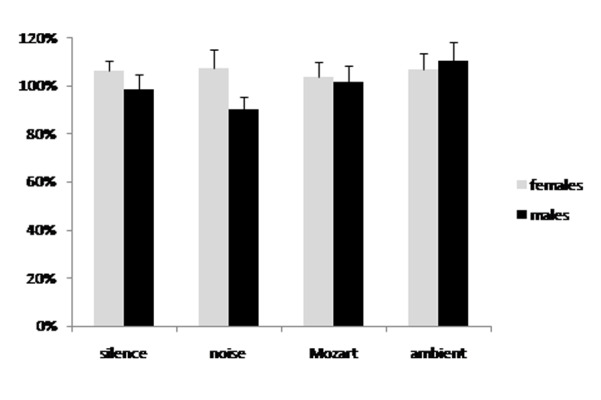
Minimal pain threshold changes due to listening to either silence, white noise, Mozart’s sonata K448 or Brian Eno’s ambient music. Data are expressed as percent of the basal values taken before listening to the recording. There was no significant difference between the groups and all groups were not significantly different from the basal value (100%).

**Fig. 2:**
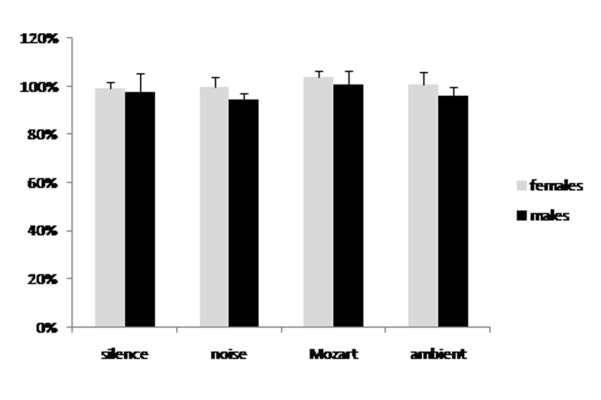
Maximal pain threshold changes due to listening to either silence, white noise, Mozart’s sonata K448 or Brian Eno’s ambient music. Data are expressed as percent of the basal values taken before listening to the recording. There was no significant difference between the groups and all groups were not significantly different from the basal value (100%).

No significant effect on pain threshold was obtained with any of the soundtrack compared to silence. In fact, listening to any of the four recordings (silence, noise, Mozart music, ambient music) resulted in a minimal (fig. 1) or maximal pain threshold (fig. 2) close to the 100% of the basal value for all groups, meaning that there was no significant effect on pain threshold. The ANOVA did not show any significant difference between the experimental groups.

## IV. DISCUSSION

The results of the present experiment did not demonstrate any effect for the soundtracks used on pain threshold. Since the present data were obtained using autoalgometry, it can be argued that music does not affect pain threshold and that the positive effects of music on pain described in literature [16–18] could be ascribed to a psychological effect, meaning that music could affect mood and, by this way, the personal feeling and evaluation of the discomfort evoked by a particular painful stimulus. A recent meta-analysis showed that lot of studies reported a positive effect of music on pain management, without a significant association with music characteristics [22]; this observation is consistent with a nonspecific effect of music, not related to the physical properties of music and, thus, not related to a simple sensory stimulation. For this reason, music will not affect sensory mechanisms, including nociceptor transmission as confirmed by the present study.

In previous experiments it has been observed that the minimal and the maximal pain thresholds can be differently affected by a pathological condition or by a treatment [19, 20, 21]. The minimal pain threshold is supposed to be mainly influenced by the actual mechanical threshold for nociceptor activation, while the maximal pain threshold is supposed to be influenced by central mechanisms of pain transmission and modulation.

This interpretation was suggested by the observation that auricular acupressure (which is supposed to act mainly on central mechanisms of pain modulation) reduced the maximal but not the minimal pain threshold [21].

The lack of an effect for any of the soundtracks used in the present study on both the minimal and the maximal pain threshold can mean that music does not affect neither nociceptor activation nor the transmission of pain from primary afferent to other central neurons. On the other hand, it is evident that music can have an effect on mood.

In fact, there are many papers reporting the efficacy of music in mood improvement in different pathological conditions [24–26] and with possible associated positive effects on pain perceptions [27, 28].

It must be considered that the lack of effects on pain threshold can be ascribed to the method used, which consisted of listening to music or noise for 4 min before and during the autoalgometric test. Thus it can be argued that music does not have an acute effect on pain threshold. Other (longer) auditory stimulations should be evaluated in future studies for possible effects on pain threshold.

Moreover, the participants to this study were not characterized from a psychological point of view; such a preliminary evaluation should be considered in future studies.

## References

[1] Bardoni R (2019). Serotonergic modulation of nociceptive circuits in spinal cord dorsal horn. Curr Neuropharmacol.

[2] Zubrzycki M, Stasiolek M, Zubrzycka M (2019). Opioid and endocannabinoid system in orofacial pain. Physiol Res.

[3] Viggiano A, Monda M, Viggiano A, Chiefari M, Aurilio C, De Luca B (2004). Evidence that GABAergic neurons in the spinal trigeminal nucleus are involved in the transmission of inflammatory pain in the rat: a microdialysis and pharmacological study. Eur J Pharmacol.

[4] Viggiano E, Monda M, Viggiano A, Viggiano A, Aurilio C, De Luca B (2010). Persistent facial pain increases superoxide anion production in the spinal trigeminal nucleus. Mol Cell Biochem.

[5] Viggiano A, Monda M, Viggiano A, Viggiano D, Viggiano E, Chiefari M, Aurilio C, De Luca B (2005). Trigeminal pain transmission requires reactive oxygen species production. Brain Res.

[6] Turner RP (2004). The acute effect of music on interictal epileptiform discharges. Epilepsy Behav.

[7] Lin LC, Lee WT, Wu HC, Tsai CL, Wei RC, Jong YJ (2010). Mozart K.448 and epileptiform discharges: effect of ratio of lower to higher harmonics. Epilepsy Res.

[8] Lin LC, Lee WT, Wu HC, Tsai CL, Wei RC, Mok HK (2011). The long-term effect of listening to Mozart K.448 decreases epileptiform discharges in children with epilepsy. Epilepsy Behav.

[9] Hughes JR, Fino JJ, Melyn MA (1999). Is there a chronic change of the “Mozart effect” on epileptiform activity? A case study. Clin Electroencephalogr.

[10] Lahiri N, Duncan JS (2007). The Mozart effect: encore. Epilepsy Behav.

[11] Miranda M, Kuester G, Ríos L, Basaez E, Hazard S (2010). Refractory non convulsive status epilepticus responsive to music as an add-on therapy: a second case. Epilepsy Behav.

[12] Lin LC, Lee WT, Wang CH, Chen HL, Wu HC, Tsai CL (2011). Mozart K.448 acts as a potential add-on therapy in children with refractory epilepsy. Epilepsy Behav.

[13] Bodner M, Turner RP, Schwacke J, Bowers C, Norment C (2012). Reduction of seizure occurrence from exposure to auditory stimulation in individuals with neurological handicaps: a randomized controlled trial. PLoS One.

[14] Coppola G, Toro A, Operto FF, Ferraioli G, Pisano S, Viggiano A, Verrotti A (2015). Mozart’s music in children with drug-refractory epileptic encephalopathies. Epilepsy & Behavior.

[15] Coppola G, Operto FF, Caprio F, Ferraioli G, Pisano S, Viggiano A, Verrotti A (2018). Mozart’s music in children with drug-refractory epileptic encephalopaties: Comparison of two protocols. Epilepsy & Behavior.

[16] Shabani F, Nayeri ND, Karimi R, Zarei K, Chehrazi M (2016). Effects of music therapy on pain responses induced by blood sampling in premature infants: a randomized cross-over trial. Iranian Journal of Nursing and Midwifery Research.

[17] Bates D, Bolwell B, Majhail NS, Rybicki L, Yurch M, Abounader D, Kohuth J, Jarancik S, Koniarczyk H, McLellan L, Dabney J, Lawrence C, Gallagher L, Kalaycio M, Sobecks R, Dean R, Hill B, Pohlman B, Hamilton BK, Gerds AT, Jagadeesh D, Liu HD (2017). Music therapy for symptom management after autologous stem cells transplantation: results from a randomized study. Bio Blood Marrow Transplant.

[18] Bashiri M, Akçali D, Coşkun D, Cindoruk M, Dikmen A, Çifdalöz BU (2018). Evaluation of pain and patient satisfaction by music therapy in patients with endoscopy/colonoscopy. Turk J Gastroenterol.

[19] Viggiano A, Zagaria N, Passavanti MB, Pace MC, Paladini A, Aurilio C, Tedesco MA, Natale F, Calabrò R, Monda M, De Luca E (2009). New and low-cost auto-algometry for screening hypertension-associated hypoalgesia. Pain Pract.

[20] Viggiano A, Passavanti MB, Zagaria G, Pace MC, Giordano M, Esposito F (2015). Anti-hypertensive treatments and hypertension-associated hypoalgesia evaluated by auto-algometry. Journal of Anesthesia and Clinical Research.

[21] Santoro A, Nori SL, Lorusso L, Secondulfo C, Monda M, Viggiano A (2015). Auricular acupressure can modulate pain threshold. Evid Based Complement Alternat Med.

[22] Martin-Saavedra JS, Vergara-Mendez LD, Pradilla I, Vélez-van-Meerbeke A, Talero-Gutiérrez C (2018). Standardizing music characteristics for the management of pain: A systematic review and meta-analysis of clinical trials. Complement Ther Med.

[23] Lorusso L, Salerno M, Sessa F, Nicolosi D, Longhitano L, Loreto C, Carotenuto M, Messina A, Monda V, Villano I, Cibelli G, Valenzano A, Monda M, Murabito P, Mollica MP, Messina G, Viggiano A (2018). Autoalgometry: an important tool for pressure pain threshold evaluation. J Clin Med.

[24] Dóro CA, Neto JZ, Cunha R, Dóro MP (2017). Music therapy improves the mood of patients undergoing hematopoietic stem cells transplantation (controlled randomized study). Support Care Cancer.

[25] Baylan S, Swann-Price R, Peryer G, Quinn T (2016). The effects of music listening interventions on cognition and mood post-stroke: a systematic review. Expert Rev Neurother.

[26] Raglio A, Attardo L, Gontero G, Rollino S, Groppo E, Granieri E (2015). Effects of music and music therapy on mood in neurological patients. World J Psychiatry.

[27] Fernando GVMC, Wanigabadu LU, Vidanagama B, Samaranayaka TSP, Jeewandara JMKC (2019). “Adjunctive Effects of a Short Session of Music on Pain, Low-mood and Anxiety Modulation among Cancer Patients” - A Randomized Crossover Clinical Trial. Indian J Palliat Care.

[28] Xue F, Landis R, Wright SM (2018). Playing Music for Hospitalized Patients Enhances Mood and Reduces Perceptions of Pain. South Med J.

